# Mining clinical attributes of genomic variants through assisted literature curation in Egas

**DOI:** 10.1093/database/baw096

**Published:** 2016-06-07

**Authors:** Sérgio Matos, David Campos, Renato Pinho, Raquel M. Silva, Matthew Mort, David N. Cooper, José Luís Oliveira

**Affiliations:** ^1^IEETA/DETI, University of Aveiro, Aveiro, 3810-193, Portugal; ^2^BMD Software, Aveiro, Portugal; ^3^Department of Medical Sciences, iBiMED, University of Aveiro, Aveiro, 3810-193, Portugal; ^4^Institute of Medical Genetics, Cardiff University, Heath Park, Cardiff, UK

## Abstract

The veritable deluge of biological data over recent years has led to the establishment of a considerable number of knowledge resources that compile curated information extracted from the literature and store it in structured form, facilitating its use and exploitation. In this article, we focus on the curation of inherited genetic variants and associated clinical attributes, such as zygosity, penetrance or inheritance mode, and describe the use of Egas for this task. Egas is a web-based platform for text-mining assisted literature curation that focuses on usability through modern design solutions and simple user interactions. Egas offers a flexible and customizable tool that allows defining the concept types and relations of interest for a given annotation task, as well as the ontologies used for normalizing each concept type. Further, annotations may be performed on raw documents or on the results of automated concept identification and relation extraction tools. Users can inspect, correct or remove automatic text-mining results, manually add new annotations, and export the results to standard formats. Egas is compatible with the most recent versions of Google Chrome, Mozilla Firefox, Internet Explorer and Safari and is available for use at https://demo.bmd-software.com/egas/.

Database URL: https://demo.bmd-software.com/egas/

## Introduction

Biological and biomedical data are continually shared through the scientific literature. Structuring this vast and ever increasing amount of information into reference resources, where it can be more easily located and used, is a very challenging and expensive task. Text-mining (TM) tools are therefore becoming a normal part of the curation pipeline, helping to expedite the work of curation teams (1–3). Nonetheless, there is still a gap between the biocuration and the biomedical TM communities, in part owing to the complexity and specific requirements of each curation task. It is important to provide expert curators with tools that take advantage of automatic TM pipelines and of existing knowledge resources, but in order to effectively support users in their daily activities, these tools must be supported by interactive and highly usable interfaces that are adapted to the curation task (4).

Following this demand, several biocuration tools have been proposed, many of which have been fostered by the BioCreative Interactive Annotation Task series (http://www.biocreative.org/tasks/biocreative-v/track-5-IAT/), which has stimulated the interaction between biocurators, text miners and tool developers, validating the positive impact of assisted curation tools and promoting their adoption. Brat (5) is one of the most popular of these tools. Brat supports in-line annotation on documents and provides concept normalization features, integration of automatic annotation services, search capabilities and document comparison. However, configuring the target concepts and relations, normalization resources and automatic services is only accessible to advanced users. Additionally, document representation may become slow when displaying full-text documents with many annotations. The tagtog annotation tool (6) also offers automatic annotation services, in addition to manual editing, for a set of pre-defined concepts. This system uses the revisions made by users to update the machine-learning annotation models, thereby improving accuracy based on user inputs. MyMiner (7) is another complete web-based solution for biocuration, supporting document triage, automatic concept recognition and document comparison. However, interpreting the annotations and their textual context is less immediate, since annotations are shown on a table rather than through in-line markup. Argo (8) offers a different approach, allowing users to design their own text analysis workflow based on integrated TM components. Thus, users are able to create custom processing pipelines for concept and relation annotation, and manually curate the results of their analysis. Even though this approach is powerful, providing a high level of flexibility, creating such workflows may require advanced expertise and a steep learning curve for biocurators. Other solutions, such as BioQRator (9), PubTator (10), RLIMS-P (11, 12) and Ontogene (13) present typical web-based interfaces with tabular listings of concept and/or relation annotations with simple highlighting and sorting capabilities. Nonetheless, some of those solutions incorporate interesting features. For instance, BioQRator integrates document triage for protein–protein interactions, PubTator features a PubMed-like interface and integrates many state-of-the-art automatic solutions for concept recognition and normalization. RLIMS-P is a more specific tool, designed to extract protein phosphorylation information using carefully designed rules and patterns for identifying concepts and relations. Neves and Leser (14) present a comparative analysis of thirteen text annotation and curation tools, based on 35 criteria; these authors concluded that although comprehensive and easy-to-use solutions exist for many use cases, no tool fully satisfied all initial criteria. The major distinguishing aspects identified by the authors were the types of annotation supported by the tool and if these are configurable, import of pre-annotated documents and integration of automatic annotation methods, support for larger annotation projects and calculation of inter-annotator agreement (IAA).

In this article, we focus on the task of identifying mentions of human genomic variants in the biomedical literature, and associating these mentions to corresponding genes, phenotypes and clinical attributes such as mode of inheritance, penetrance and zygosity. To support this task, we propose Egas, a project-oriented and customizable web platform for TM assisted literature curation that offers manual and automatic annotation of concepts and concept relations, with simple in-line representations and straightforward user interactions. Egas provides ‘annotation-as-a-service’ through a centrally managed pipeline, including document collections, users, configurations, annotations, back-end data storage and document processing and TM tools (15). Its flexible architecture, allowing the definition of concept and relation types to use on each annotation project, integration of automatic annotation web-services and the ability to import pre-annotated documents in different formats (e.g. the A1 standoff annotation (16) and BioC inline XML format (17)) facilitates adaptation to different annotation requirements. Additionally, several ontologies from NCBO BioPortal (http://bioportal.bioontology.org/) are integrated and can be configured as the normalization ontology to use for each concept type. These features give curation teams the practicality and simplicity of using an annotation service configured according to each project’s annotation guidelines.

## Curation of Genomic Variant Clinical Attributes

The study of genetic variations and their association with diseases is a major focus of biomedical research (18). Several databases have been set up to curate and store these associations and related information in structured form, but automated methods to extract this information from text are essential in order to keep track of the burgeoning biomedical literature. This has prompted the development of various TM tools for identifying genetic variants in biomedical texts. Wei *et al.* (19) described tmVar, a conditional random field (CRF)-based variant extraction tool together with a corpus of 500 Medline abstracts used for training and testing the models. The authors compared their model to the rule-based system, MutationFinder (20), and showed significant improvements in terms of recognition performance when tested in their corpus as well as on the MutationFinder corpus. tmVar uses an extensive feature set and a specially crafted set of labels that allows the CRF model to identify different variation types. Additionally, a small set of post-processing rules is applied to further improve the model predictions, leading to a state-of-the-art F-score of 91.4% (87.7% when no post-processing was applied), an increase of over 13 percentage points when compared with MutationFinder. Other studies have considered not only the identification of variant mentions but also the association with disease. Doughty *et al.* (21) used their own rule-based variant extraction tool, EMU, to identify variant mentions in two sets of abstracts related to prostate cancer and breast cancer, respectively. They manually validated 51 mutations related to prostate cancer and 128 mutations related to breast cancer that had not yet been annotated in large reference databases. Ravikumar *et al.* (22) used MutationFinder to identify variants in Medline abstracts, and associated these with automatically annotated disease and protein mentions by using the dependency graph of sentences containing these three entities. They obtained an *F*-score of 64.3% when comparing to gold-standard data from the UniProt database.

Our curation task involved annotating mentions of human inherited pathogenic gene variations in Medline abstracts, as well as association to genes, diseases and clinical attributes such as inheritance mode and penetrance. The task was organized in concert with the Human Gene Mutation Database (HGMD), a comprehensive collection of germline mutations in nuclear genes that underlie, or are associated with, human inherited disease (23). By March 2016, this database contained over 183 000 different lesions detected in over 7000 different genes, with new mutation entries currently accumulating at a rate exceeding 12000 per annum. HGMD is used as a central unified disease-oriented mutation repository by human molecular geneticists, genome scientists, molecular biologists, clinicians and genetic counsellors as well as by those specializing in biopharmaceuticals, bioinformatics and personalized genomics. The public version of HGMD (http://www.hgmd.org) is freely available to registered users from academic institutions/non-profit organizations whilst the subscription version (HGMD Professional) is available to academic, clinical and commercial users under license via Qiagen Inc (http://www.biobase-international.com/product/hgmd).

The annotation task was performed on a corpus of 100 Medline abstracts selected after prioritizing, using a classifier trained with information from the documents previously used to curate information in HGMD, the 28000 results obtained from the PubMed search:

genetic disease, inborn[MeSH Terms] AND (polymorphism, genetic[MeSH Terms] OR deletion[Title/Abstract] OR substitution [Title/Abstract] OR insertion[Title/Abstract] OR duplication[Title/Abstract] OR indel[Title/Abstract] OR delin[Title/Abstract] OR conversion[Title/Abstract] OR translocation[Title/Abstract] OR inversion [Title/Abstract]) AND hasabstract[text] AND humans[MeSH Terms] AND English[lang] NOT Review[ptyp] NOT “genome wide”

Table 1 lists the concepts defined for the task, where variations were subdivided into single nucleotide variants (SNVs), insertion/deletions (InDel) and rearrangements. Automatic entity recognition tools were used to identify mentions of these concepts, in order to facilitate and accelerate the curator’s work, but the automatic annotation of relations (e.g. between a variant and a gene or disease mention) was not considered. In order to ground the annotations, concepts were assigned a concept identifier from an established ontology. The following terminologies were selected for grounding the concept annotations in this corpus:

**Table 1.** Concepts for curation task

Group	Concept
Gene	Gene
Disease	Disease
Variation	SNV
	InDel
	Rearrangement
Clinical attributes	Mode of inheritance
	Penetrance
	Zygosity
	Age of patient (years)
	Age of onset (years)

- Diseases—Online Mendelian Inheritance in Man (OMIM)
- Genes (or proteins)—HUGO Gene Nomenclature Committee (HGNC)
- SNVs—dbSNP

OMIM and HUGO are part of the various vocabularies available in Egas for concept normalisation. The dbSNP data, on the other hand, were specially collected and integrated for this annotation task. This required indexing all variants available in the dbSNP database, together with all Human Genome Variation Society names associated with each variant as well as the corresponding gene names, to facilitate matching the textual mention to an existing dbSNP identifier. Normalization of insertion-deletions and rearrangements was not considered.

To annotate the clinical attributes, we collected terms (and their synonyms) from OMIM, Human Phenotype Ontology, NCBI Metathesaurus, and NCI Thesaurus. A small ontology was created using the selected terms, and integrated in Egas. Table 2 lists the set of clinical attributes considered for this task and the Unified Medical Language System (UMLS) concept unique identifiers (CUI) used to represent the categorical values of these attributes. All synonyms for these terms were compiled in a dictionary for automatic matching in the texts.

**Table 2.** List of clinical attributes considered for annotating human variants

Concept	Clinical metadata	UMLS CUI
**Mode of inheritance**	Autosomal dominant	**C0265385**
	Autosomal recessive	**C0441748**
**Zygosity**	Homozygous	**C0019904**
	Heterozygous	**C0019425**
	Hemizygous	**C1881036**
**Penetrance**	Complete penetrance	**C1840470**
	Reduced penetrance	**C1867989**
	Variable penetrance and expressivity	**C3276568**
	Incomplete penetrance of some features	**C2750454**
	Incomplete, age-associated penetrance	**C3280136**
**Age**	Age of patient (years)	**C0001779**
	Age of onset (years)	**C0206132**

## Curation Tool

**Figure 1.**
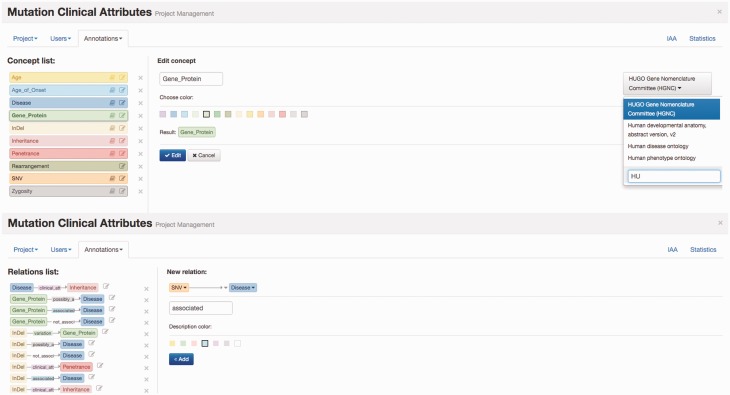
Egas administration panel illustrating the definition of concept type ‘Gene_Protein’ linked to HGNC for normalization, and of the ‘associated’ relation between ‘SNV’ and ‘Disease’ concepts.

Egas organizes curation tasks in projects. Each project created in Egas has its own workspace, and comprises a curation or document annotation task, performed on a collection of documents, by a team of (one or more) curators, and considering a pre-defined set of concept and relation types defined by the curation guidelines. Project administrators have access to the administration panel that allows them to add and manage users (curators) associated with the project, import documents, and define project characteristics and annotation guidelines. The project administrator can freely define the relevant concept and relation types and the normalization ontology to use for each concept type, according to the requirements of the task. Furthermore, to facilitate the annotation work, each different concept and relation type may be associated with a markup colour (Figure 1) .

**Figure 2.**
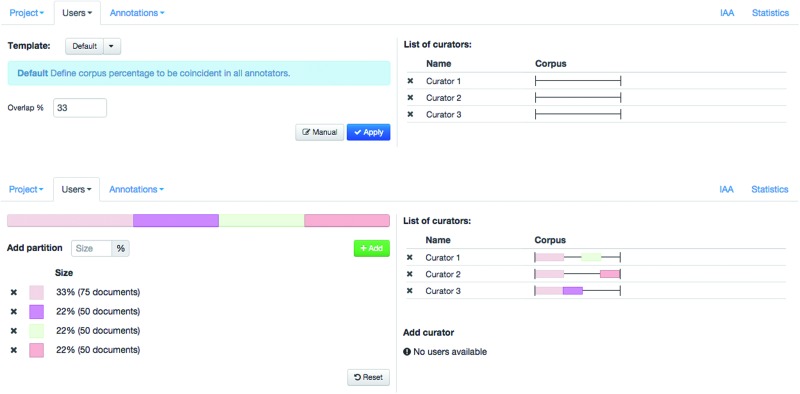
Administration interface showing the definition and assignment of corpus partition to curators.

Egas supports collaborative and blind annotation projects. In the case of collaborative projects users can work on the same documents, see the changes introduced by other users, and use the project chat to discuss details of the annotation task, contributing to more consistent results. Blind annotation projects allow project administrators to assign different portions of the corpus to different users, with a configurable overlap. Administrators may evaluate the annotation consistency by visually comparing the annotations or by calculating the IAA (see ‘Results’ section). Figure 2 shows the ‘Users’ tab in the user management panel, where the project administrator can partition the corpus and assign partitions to curators. Partitions may be created manually, by defining their size in terms of proportion of the corpus, or a simple overlapping schema can be used. In this case, the administrator only needs to define the percentage of overlap between curators, and the tool generates and assigns the partitions according to the number of curators in the project. Any remaining documents are equally distributed among curators. In the example shown, an overlap of 33% is defined and each of the three curators is assigned a shared partition comprising 33% of the documents in the corpus, plus an individual partition with 22% of the corpus. To facilitate assigning or removing a partition previously assigned to a curator, the coloured partition boxes may be dragged over to (or from) a curator on the right-hand side of the panel.

**Figure 3.**
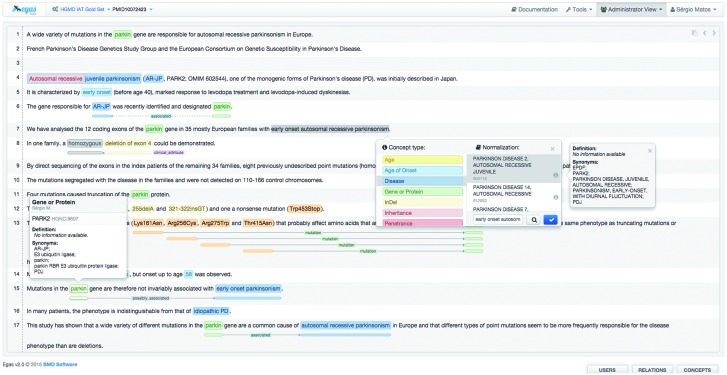
Egas annotation interface illustrating the addition of a new concept annotation of type ‘Disease’, and its normalization to an OMIM concept. A concept information tooltip is shown when hovering the mouse pointer over an existing annotation.

Figure 3 illustrates the annotation panel in Egas. The central box displays the content of the text being curated, showing the concepts and relations that have been identified. Concepts are shown as coloured boxes, using the colours defined in the project configuration. Hovering the mouse over an annotation reveals the corresponding semantic type and normalization information. Relations are shown as lines, tagged with the relation type. Coloured boxes connected by the relation markup are placed under the concepts that participate in the relation, making it easy to identify the entire relation.

### Assisted curation

The curation work in Egas may start with raw texts or with pre-processed texts, containing automatically identified concepts and relations that will be revised by the curators. This can be achieved by importing a previously processed document collection in either A1 or BioC formats, or by using concept and/or relation extraction web-services to annotate a set of documents in the collection. Likewise, annotated **d**ocuments can be exported in A1 or BioC formats, allowing users to store the generated information locally in order, for instance, to add it to a local knowledgebase or for use in TM pipelines.

For this task, documents were pre-annotated with Neji (24), a biomedical concept annotation framework, and imported to Egas in A1 format. Genes and SNVs were identified through CRF machine-learning models, whereas mentions of diseases and variant’s clinical attributes were identified through dictionary-matching using terms from the UMLS ‘Disorders’ semantic group and from the clinical attributes ontology described earlier. The dictionary-based annotation of diseases achieved an *F*-score of 85.0% (24) when evaluated on the NCBI Disease corpus (25). The gene recognition model was trained and evaluated on the BioCreative II Gene Mention corpus (26), and achieved an *F*-score of 87.5% on the test set of that corpus (27). For SNVs, we used Neji and the tmVar corpus to train our own CRF model, achieving an *F*-score of 86.0%.

## Results

Seven curators were asked to annotate documents that were pre-analyzed by the automatic concept recognition tool (half of the corpus), and raw documents (the remaining corpus), in order to evaluate the added benefit of TM-assisted curation. Curators had to revise the automatically generated annotations, correct any erroneous concept annotations and add missing ones, normalize the concept mentions, and add associations between the identified concepts. For the raw documents, curators had to add all the concept, normalization and relation annotations. The tool recorded the time taken by each curator to curate each document, as well as the number of annotated concepts and relations.

Three curators annotated the complete corpus while two other curators followed a time-limited work plan, that is, they worked on the curation task for a total of four hours, half of the time curating pre-annotated documents and the other half working on raw (non-annotated) documents. The remaining two curators annotated a small portion of the corpus: 13 and 9 documents.

**Figure 4.**
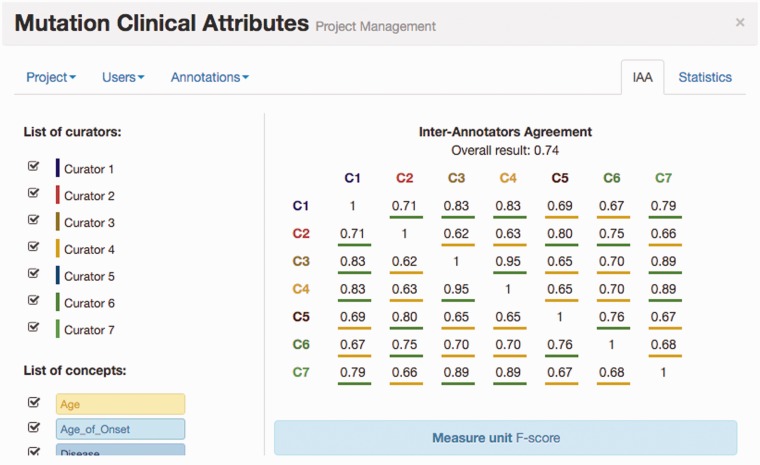
IAA panel in Egas. The IAA is calculated as the *F*-score between each pair of curators, and the average of these values is taken as the overall result. Annotations for each concept type and each curator can be included or removed from the calculation by using the checkboxes on the left.

Figure 4 illustrates the IAA panel in Egas. The IAA is calculated as the average of f-scores between each pair of curators, taking into account the documents shared by both. For this task, the complete corpus was assigned to all curators (100% overlap) in order to maximize the number of shared documents. An overall IAA of 0.74 was obtained, with paired agreements varying between 0.62 and 0.95. The IAA panel allows the project manager to obtain the agreement for each pair of curators, for all the concept types or by selecting the concept types to consider.

**Figure 5.**
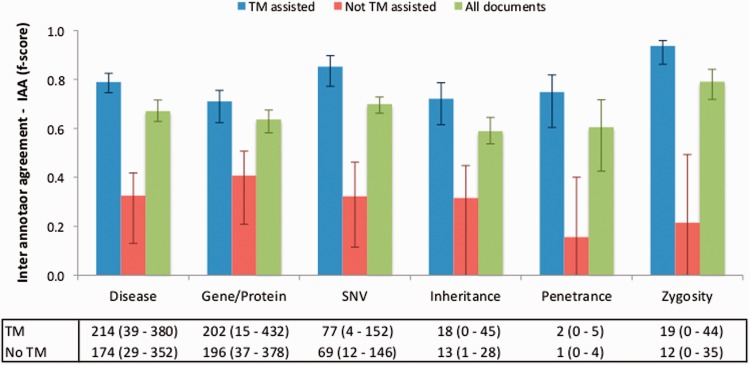
IAA obtained on documents pre-annotated with automated TM services, without pre-annotation, and on all documents. The error lines show the minimum and maximum IAA values for all pairs of annotators. The data table shows the average (range) number of annotations for each concept type using TM-assisted vs. not TM-assisted curation.

**Figure 6.**
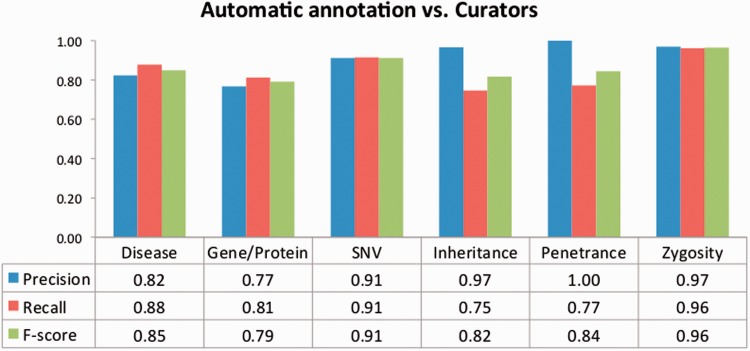
Evaluation of the automatic annotations provided in the corpus. The data table shows the average precision, recall and *F*-score against all curators.

To evaluate the impact of the TM-assisted workflow, we compared the IAA obtained on the pre-annotated documents against the agreement obtained on documents without automatic annotations and on all documents in the corpus. IAA was significantly higher when performing the curation task on pre-annotated documents than on documents without automatic annotations (*P*-value ≪ 0.01, one-tailed paired *t*-test). The results shown in Figure 5 indicate that annotation consistency can be enhanced through the use of automated TM services. This improvement may possibly be explained by the added simplicity of checking and correcting existing annotations when compared with manually adding all annotations, and by the fact that the pre-annotation promotes completeness, that is, curators are encouraged to perform a more comprehensive annotation of the document. Annotations of ‘Age’, ‘Age of Onset’, ‘InDel’ and ‘Rearrangement’ were not considered for these results, as these were only present on a very small number of documents. The accuracy of the automatic annotations could be evaluated by comparison to the results provided by the curators. Figure 6 shows average evaluation metrics of automatic annotations against all curators, with precision ranging from 0.77 for gene to 1.0 for penetrance and recall ranging from 0.75 for inheritance to 0.96 for zygosity. These results indicate the quality of annotations provided and further support the effectiveness of TM-assisted literature curation.

We also evaluated the number of annotations and the time taken to perform the task on each set of documents. As can be seen from the results in Table 3, there are in general more concept annotations in documents that had been previously annotated by the concept recognition tool and, on average, less time is required to add annotations to these documents than to documents that had not been automatically annotated. Although no relation extraction tool was used to pre-annotate the corpus, the results indicate that automatic concept recognition may also help increasing the number of relations annotated. Two of the seven curators were not considered for these results, as they only curated 9 and 13 documents, respectively.

**Table 3.** Comparison of TM-assisted versus non-assisted curation

	TM assisted	Non-assisted	*P*-value
Concepts	744	664	0.12
Relations	217	157	0.10
Concepts/article	23.5	17.4	0.13
Relations/article	6.8	4.2	0.12
Time/article	219	245	0.25
Time/concept	10.8	13.1	0.17
Time/relation	44.3	56.1	0.13

An automatic tool was used to annotate concepts but not relations. Values shown are averages for five curators. *P*-value obtained using a one-tailed paired *t*-test.

## Conclusion

We describe the use of Egas, a configurable web-based document annotation and curation tool, on a literature curation task focused on the annotation of mentions of human inherited pathogenic gene variations and their association with genes, diseases and clinical attributes. The tool allows teams of curators to work on a shared curation project, following a set of configurable concept and relation types. The curation task can be performed over a collection of raw text documents or by reviewing automatic concept and relation annotations, obtained either with the included concept and relation identification service or through external annotation tools.

To validate Egas and its assisted curation features, we participated in the BioCreative Interactive Annotation task, obtaining positive evaluation in terms of usability, learnability and design on a user survey conducted by the task organizers (28). Additionally, we analysed the IAA and time spent when annotating raw documents and when working on documents previously annotated by an automatic concept recognition tool. The survey results and our comparative analysis show that a TM-assisted curation pipeline brings benefits in terms of efficiency and consistency of the curation results.
